# Navigating the Metaverse: A New Virtual Tool with Promising Real Benefits for Breast Cancer Patients

**DOI:** 10.3390/jcm13154337

**Published:** 2024-07-25

**Authors:** Weronika Magdalena Żydowicz, Jaroslaw Skokowski, Luigi Marano, Karol Polom

**Affiliations:** 1 Department of General Surgery and Surgical Oncology, “Saint Wojciech” Hospital, “Nicolaus Copernicus” Health Center, Jana Pawła II 50, 80-462 Gdańsk, Poland; veronikazydowicz@wp.pl (W.M.Ż.); j.skokowski@amisns.edu.pl (J.S.); 2 Department of Medicine, Academy of Applied Medical and Social Sciences, Akademia Medycznych I Spolecznych Nauk Stosowanych (AMiSNS), 2 Lotnicza Street, 82-300 Elbląg, Poland; k.polom@amisns.edu.pl; 3 Department of Gastrointestinal Surgical Oncology, Greater Poland Cancer Centre, Garbary 15, 61-866 Poznan, Poland

**Keywords:** metaverse, virtual reality, AR, BC, breast surgery

## Abstract

**Simple Summary:**

This research explores how virtual worlds like the Metaverse can improve breast cancer (BC) diagnosis and treatment. The authors aim to show how these virtual platforms can simulate operations, provide patient support, and facilitate research, ultimately making healthcare more efficient and effective. The findings suggest that the Metaverse offers promising opportunities for healthcare professionals to engage with patients, leading to better outcomes and reduced costs. This research could revolutionize how BC is approached, potentially improving patient care and outcomes.

**Abstract:**

BC, affecting both women and men, is a complex disease where early diagnosis plays a crucial role in successful treatment and enhances patient survival rates. The Metaverse, a virtual world, may offer new, personalized approaches to diagnosing and treating BC. Although Artificial Intelligence (AI) is still in its early stages, its rapid advancement indicates potential applications within the healthcare sector, including consolidating patient information in one accessible location. This could provide physicians with more comprehensive insights into disease details. Leveraging the Metaverse could facilitate clinical data analysis and improve the precision of diagnosis, potentially allowing for more tailored treatments for BC patients. However, while this article highlights the possible transformative impacts of virtual technologies on BC treatment, it is important to approach these developments with cautious optimism, recognizing the need for further research and validation to ensure enhanced patient care with greater accuracy and efficiency.

## 1. Introduction

The Metaverse, a three-dimensional digital space facilitated by virtual reality (VR) and augmented reality (AR) headsets, offers users the opportunity to create avatars and access immersive experiences through stable internet connections. With unlimited access to various online platforms, the Metaverse has emerged as a transformative force with vast potential to revolutionize healthcare systems [1]. As the Metaverse continues to evolve, it presents numerous opportunities to enhance BC diagnosis and management. Patients can plan their treatment based on their avatars, which encapsulate their medical history, current health status, and specific diseases [2]. Moreover, the Metaverse serves not only as an informational resource about breast cancer (BC) but also as a global platform for doctors to collaborate on patient cases, transcending geographical barriers and time constraints. Through computer or headset software, physicians can convene virtually to discuss cases and participate in training sessions, including simulated operations [3]. This immersive training environment enables doctors to acquire new skills and refine their practice, ultimately improving medical care for BC patients. As the healthcare landscape continues to evolve, there is a growing need for platforms that uphold standards of medical care. The Metaverse emerges as a promising solution, offering personalized therapy options for BC patients. This narrative review aims to provide an overview of the Metaverse’s impact on BC care. Subsequent sections will delve into the potential risks and benefits associated with Metaverse integration, supported by an in-depth analysis of relevant data.

## 2. Materials and Methods

Our methodology involved conducting a systematic literature search across PubMed and various scientific databases. Employing a predefined set of keywords including “metaverse”, “virtual reality”, “AR”, “breast surgery”, and “BC”, we meticulously explored studies published between 2003 and 2023. Moreover, we conducted an extensive search on Google (Google LLC., Menlo Park, CA, USA, 2023) to gather information on the “Metaverse”, recognizing it as an emerging technology that has received limited attention in the literature indexed on PubMed. This approach facilitated the retrieval of a broad spectrum of relevant literature pertaining to the Metaverse’s role in BC management. By synthesizing the gathered information, we meticulously retrieved the most pertinent data directly related to the Metaverse and its implications for BC diagnosis and treatment. Through an exhaustive examination of the identified studies, we systematically analyzed their findings to draw conclusions on how the Metaverse holds the potential to revolutionize BC management. 

## 3. Metaverse and Its Influence on BC Operations

The Metaverse shows promise for potentially influencing the healthcare sector, including its potential applications in BC therapy, which may offer avenues for improved efficiency and precision. To elucidate the advantageous impact of the Metaverse on healthcare, we categorized the gathered information into subgroups to demonstrate its potential influence.

### 3.1. MeTAI

The integration of medical technology and artificial intelligence, commonly known as MeTAI, holds significant promise for transforming healthcare through the immersive and interactive capabilities of the metaverse [4,5,6]. This fusion not only enhances existing interventions but also sparks the creation of innovative methodologies in medical research, training, and patient care [7]. MeTAI represents a shift from traditional Electronic Medical Record (EMR) systems by utilizing immersive VR technology, revolutionizing the visualization, accessibility, and analysis of medical data [8]. For instance, MeTAI can systematically generate comprehensive datasets from patients, their avatars, and phantoms, seamlessly integrating and disseminating this information [8,9]. Moreover, it can harmonize datasets to facilitate multitasking and systems biomedicine [10,11]. One primary advantage of MeTAI is its ability to support virtual clinical trials and simulations, overcoming the logistical and ethical challenges of traditional methods [12]. AI-driven recruitment, real-time progress monitoring, and data analysis accelerate the development and refinement of medical interventions, ensuring their efficacy and safety before widespread implementation [13,14]. An early validation of MeTAI’s efficacy is seen in the FDA-led Virtual Imaging Clinical Trial for Regulatory Evaluation (VICTRE) study [15]. This project involved creating 2986 in silico patients to evaluate the efficacy of digital breast tomosynthesis (DBT) as a surrogate for full-field digital mammography in breast cancer screening. The simulation, covering the entire imaging continuum from virtual patients to 2D and 3D X-ray mammographic images, yielded results consistent with those from a parallel human trial. These findings were submitted to the FDA as part of a pre-market application for DBT device approval, underscoring the potential of simulation tools in regulatory assessments. Additionally, the FDA established the “Medical Device Development Tools” (MDDT) program, which qualifies non-clinical assessment models, including animal and computational models, and datasets as MDDTs [16]. One of MeTAI’s most profound impacts is its potential to revolutionize the utilization of medical data and tools by practitioners and patients, facilitating screening and diagnosis, understanding diseases, selecting therapies, and executing precise interventions [8,11]. Enhancing lesion detection for medical professionals is a key goal of AI-based tools [17]. Recent evidence shows the benefits of integrating AI in mammography screening as a complementary diagnostic tool. Schaffter T et al. [18] highlighted that while no single AI algorithm surpassed radiologists, combining AI algorithms with radiologist assessments in single-reader screenings improved overall accuracy. A European study on 429 women with interval cancer suggested that a deep learning AI system could reduce interval cancer rates without additional screening methods [19]. Large-scale retrospective studies have supported AI’s effectiveness. For example, a European study involving 122,969 mammography examinations from 47,877 women demonstrated AI’s ability to detect screen-detected cancers at various thresholds [20]. Another study with 170,230 mammograms from institutions in South Korea, the US, and the UK showed AI’s superior diagnostic performance compared to radiologists [21]. Kim HJ et al. [22] found that AI systems significantly aided in detecting mammographically occult breast cancers, especially in dense breasts, with many being asymptomatic, invasive, and already metastasized to axillary lymph nodes. Despite these advancements, many studies are small and retrospective, indicating the need for prospective trials. The MASAI randomized controlled trial aims to assess AI’s impact on mammography screening efficacy, focusing on reducing interval cancer rates [23]. A trial randomly assigned 80,033 patients to AI-supported screening (*n* = 40,003) or double reading without AI (*n* = 40,030). The cancer detection rates were 6.1 per 1000 in the AI group (95% CI 5.4–6.9) and 5.1 per 1000 in the control group (95% CI 4.4–5.8), with a ratio of 1.2 (95% CI 1.0–1.5; *p* = 0.052). Recall rates were 2.2% in the AI group (95% CI 2.0–2.3) and 2.0% in the control group (95% CI 1.9–2.2), with both groups having a false-positive rate of 1.5% (95% CI 1.4–1.7). The use of AI reduced the screen-reading workload by 44.3%, highlighting that AI-supported mammography screening resulted in a similar cancer detection rate compared to standard double reading, with a substantially lower screen-reading workload, indicating the safety of AI use in mammography screening. Furthermore, the trial was not stopped and will continue to assess the primary endpoint of the interval cancer rate in 100,000 participants after a two-year follow-up. On the other hand, applying AI to DBT presents challenges due to its 3D nature. However, studies have shown AI’s effectiveness in identifying suspicious features in DBT images [24,25]. Van Winkel SL et al. [26] found that AI support improved radiologists’ accuracy and reading times in DBT interpretation, and Conant EF et al. [27] demonstrated that AI assistance improved radiologists’ performance in detecting malignant lesions, reducing recall rates and reading times. Validating AI systems requires large datasets, such as the one curated by Buda M et al., which includes 22,032 DBT volumes from 5060 and is available via the Cancer Imaging Archive [28]. In MRI, QuantX™ from Qlarity Imaging is the only FDA-approved CAD tool used for assessing breast abnormalities. A study showed that using QuantX™ improved radiologists’ ability to differentiate between benign and malignant lesions, increasing the average area under the curve (AUC) from 0.71 to 0.76 [29]. Furthermore, within such a virtual environment, intricate surgical and therapeutic cases can be meticulously planned and rehearsed, employing iterative trial-and-error methods in a risk-free scenario. This capability synergizes with contemporary surgical systems, exemplified by da Vinci, which facilitate remote surgical procedures through high-speed internet connectivity, thereby transcending geographical constraints. In MeTAI, surgeons can experiment with diverse approaches, such as plastic surgery, on virtual avatars [8,9]. Similarly, MeTAI expands the practice of optimizing treatment plans through patient-specific computer simulations to encompass all medical interventions [30]. Furthermore, MeTAI enables the simulation of biological responses to therapy delivery, allowing for the customization of treatment responses based on individual genetic profiles and aggregated patient data [31,32,33]. These innovations have facilitated the prediction of patient responses to novel adjuvant therapies, leading to improved treatment outcomes. The advancement of computer technology has enabled the efficient analysis of large and complex datasets through bivariate and multivariable regression calculations and modeling [34]. In a study by Hong JC et al. [35], the researchers evaluated the mean heart dose (MHD) of adjuvant radiation therapy (RT) for breast cancer and the estimated risk of RT-associated cardiotoxicity in female populations. Their findings indicated that MHD varied depending on the RT technique and was influenced by patient positioning and breathing during RT. The overall risk of cardiotoxicity was moderate, with an additional 3.5 events per 1000 patients, varying based on the employed RT technique. In another study by Sager O et al. [36], the effectiveness of adaptive radiotherapy (ART) was assessed by rescheduling the tumor bed boost using repetitive CT simulations after whole-breast irradiation (WBI) for patients with seroma. This study, which included 48 patients, formulated two RT therapeutic regimes for each patient to monitor changes in seroma and boost target volume. The results demonstrated a significant reduction in seroma volume and critical organ doses with ART, underscoring the benefits of ART in reducing seroma and critical organ doses for patients undergoing WBI. These findings hold significant implications for optimizing RT treatment planning and improving patient outcomes. High doses of radiation in radiotherapy can potentially harm the heart and blood vessels, making it crucial to carefully monitor breast cancer patients receiving radiotherapy and take measures to minimize cardiac toxicity risk. Jung JW et al. [37] developed a novel automated methodology for segmenting cardiac substructures in radiotherapy CT images. Their study revealed minimal variance in doses for simulated breast radiotherapy between automatic and manual contours. Additionally, the use of more than ten atlases did not significantly enhance performance, nor did manual guide points significantly improve the method’s efficacy. In radiation oncology, the current standard treatment approach is based on general clinical test results, which lack individuation and fail to account for individual patient responses. Integrating mathematical models into radiation oncology has the potential to improve treatment evaluation and lead to enhanced patient outcomes through individualized ART [38]. By utilizing mathematical models to simulate a patient’s tumor growth and forecast treatment response, dynamic biomarkers can be developed for RT, facilitating individualized patient treatment. Machine learning algorithms that combine imaging data, molecular data, and demographic data have been employed to predict breast cancer patients’ response to neoadjuvant chemotherapy. Duanmu H et al. [39] utilized a convolutional neural network with a novel approach that combined 3D MRI imaging data, molecular data, and demographic data to predict the probability of a pathological complete response to neoadjuvant chemotherapy in breast cancer patients. This method achieved high accuracy and AUC values, outperforming models that used only imaging data or conventional concatenation models. Byra M et al. [40] proposed a promising deep learning approach using ultrasound imaging, employing transfer learning with convolutional neural networks and comparing the results with a traditional method based on handcrafted morphological features. Their study showed promising performance, with the best model achieving an AUC of 0.847 in comparing ultrasound images before and after treatment. Additionally, Yang L et al. [41] developed a prediction model using a combination of gene expression and a machine learning algorithm, which showed significant differences in pCR rates between sensitive and insensitive groups. The Naive Bayes algorithm demonstrated the highest predictive value, with a sensitivity of 84.5 and specificity of 62%. Although current computational limitations and uncertainties in biological models hinder routine implementation, the integration of simulators and clinical knowledge repositories within the metaverse holds promise for mitigating organ damage risks as well as improving oncological outcomes. This paradigm shift promises to optimize treatment planning processes, leveraging the immediacy and accessibility afforded by virtual environments [42,43,44,45]. Furthermore, the utilization of AI-powered virtual and AR environments represents a significant advancement in educational methodologies, offering trainees enhanced learning opportunities [8]. These environments allow for the repetitive practice of simple as well as complex procedures and provide personalized feedback, thereby facilitating continuous skill refinement. This innovation has played a pivotal role in the seamless integration of novel systems into clinical settings, as evidenced by the development of surgical robotic simulators and tailored educational curricula [43]. Moreover, specific medical institutions are leading initiatives to revolutionize anatomy education by adopting VR and AR platforms, effectively reducing the need for traditional cadaver-based learning approaches [44]. Additionally, in telemedicine, MeTAI powered by AI algorithms enables the real-time analysis of patient data, providing immediate diagnostic insights and treatment recommendations [46]. This is particularly valuable in remote or underserved areas with limited access to healthcare [47]. Virtual consultations and follow-ups conducted within the metaverse maintain high levels of interaction and engagement between patients and healthcare providers, while personalized virtual environments enhance patient adherence to treatment plans [11,48]. Anyway, none of these potential benefits come without challenges. The successful implementation of the MeTAI in healthcare requires a sophisticated architecture and infrastructure that seamlessly integrates patients, physicians, researchers, algorithms, devices, and data [8]. Given the revolutionary potential of MeTAI, it is essential to anticipate and address the associated challenges proactively. These challenges include ensuring interoperability among diverse systems, safeguarding data privacy and security, and developing comprehensive training programs for practitioners [42]. By taking prompt and strategic actions, an optimal course for the development of the MeTAI metaverse can be charted, maximizing its benefits while upholding the highest standards of care and efficiency. However, these technological challenges could impact the accuracy and applicability of MeTAI in medical fields. Practitioners may initially experience stress or distraction during the early phases of MeTAI adoption [42]. As MeTAI technology develops, there will be a need for training and certification for practitioners. Surgeons and interventional radiologists might find it challenging to use new tools or robots, similar to the risks posed by new aircraft automation to untrained pilots. To aid in the introduction of new systems, surgical robotic simulators and curricula should be developed [43]. Some medical schools are now using VR and AR platforms for cadaver-less anatomy education [44]. The field of human–computer interaction in the metaverse has led to the creation of a Metaverse Knowledge Center by computer scientists (https://metaverse.acm.org/) (accessed on 2 April 2024). Companies like OSSO VR are advancing VR-based training for surgical procedures (https://www.ossovr.com/) (accessed on 2 April 2024). MeTAI facilitates collaborative and continuous learning, multi-institutional projects, team training, and co-development through realistic metaverse interactions.

### 3.2. MIoT

The term Medical Internet of Things (MIoT), introduced by Yang D et al. as a definition of the Metaverse in Medicine [11], represents the next-generation mobile computing platform that combines communication technologies with intelligent mobile devices, facilitated through AR and VR glasses. The enhancement of MIoT within the healthcare sector enables precise prediction and diagnosis of various diseases by healthcare professionals and patients through data analysis [49]. MIoT allows for remote management and sensing of data from smart health devices over network infrastructures, reducing human intervention and improving efficiency, accuracy, productivity, and cost-effectiveness. Yang D et al. [11] were pioneers in the MIoT in China and globally, developing the first MIoT-based home tele-monitoring and management platform for obstructive sleep apnea-hypopnea syndrome (OSAHS) [50]. Although MIoT is still in its early stages, it shows great potential and has been applied in various medical fields. This technology is experiencing significant growth in clinical applications and the expansion of embedded devices that integrate information and real-world objects, creating a vast healthcare market that benefits patients. The MIoT concept is increasingly recognized internationally [51]. Various studies have demonstrated its extensive influence. In the Strategy for American Innovation (2014), IT adoption in medicine and healthcare was highlighted as one of six priority fields for innovation in the USA [52]. For example, the Asthma Health App (AHA) (version 1.0) conducts large-scale health research and provides real-time air pollution monitoring, using data from users’ electronic asthma diaries to predict acute attacks and aid in disease prevention [53]. Another significant contribution comes from Intel, as part of the Leading Age Center for Aging Services Technologies (CAST), which developed wireless sensor networks (WSNs) for in-home healthcare solutions [54]. These sensors, embedded in everyday objects, help the elderly and disabled live independently while allowing medical staff and social workers to provide necessary assistance. The EU Information Society Technologies (IST) Framework Programme 5 (FP5) funded the AMON project, which developed a wearable tele-monitoring and alert system that collects and evaluates vital parameters, detects medical emergencies, and connects to medical centers via cellular networks [55]. STMicroelectronics and Mayo Clinic developed an innovative telemedicine platform for managing chronic cardiovascular diseases, offering long-term monitoring and treatment options without disrupting daily activities [56]. Japan, with its robust network and technological foundation for IoT, has been investing in medical informatization. Toshiba developed an AI system using wrist-worn sensors and a PDA to monitor and analyze health, daily activities, and habits [57]. This system offers personalized advice on diet and exercise, playing a crucial role in behavioral change and reducing lifestyle-related disease risks. It also supports BC perioperative programs by accurately tracking and collecting patients’ vital signs, response to treatment, and overall health trends, fostering more proactive and tailored approaches to BC management [58]. Particularly, the integration of MIoT into BC care opens up numerous avenues for enhancing detection, treatment, and patient support. Various studies [59,60,61,62] have explored the diverse applications of this technology in this context, emphasizing its potential to revolutionize healthcare practices. One prominent area where IoT can make a significant impact is in early detection and screening. Through the utilization of smart bras and wearables embedded with sensors, changes in breast temperature, moisture, or tissue density can be continuously monitored [63]. These alterations may serve as early indicators of breast abnormalities, prompting individuals to seek further medical evaluation. Additionally, MIoT-enabled devices can facilitate remote monitoring of mammogram results in real-time, allowing for quicker analysis by radiologists [58]. To this address, Ogundokun R et al. [49] developed an MIoT-based framework for BC diagnosis using hyperparameter-optimized neural networks. The study focuses on optimizing Convolutional Neural Network (CNN) and Artificial Neural Network (ANN) models to enhance diagnostic accuracy. It compares Support Vector Machine (SVM) and Multi-Layer Perceptron (MLP) classifiers, using Particle Swarm Optimization (PSO) for feature selection, and evaluates these models with the Wisconsin BC (WBC) dataset. The research demonstrates, over a total of 569 participants, that the MLP classifier with PSO feature selection outperforms other classifiers, and the ANN model achieves slightly higher accuracy than the CNN model. These results highlight the importance of hyperparameter optimization and feature selection in improving machine learning model performance for BC diagnosis, showcasing the potential of MIoT and advanced algorithms to improve healthcare outcomes. In terms of treatment and medication management, MIoT presents opportunities for improved adherence and personalized care. Smart drug dispensers, connected to MIoT networks, can assist patients in adhering to their prescribed treatment regimens by providing reminders and notifications to both patients and healthcare providers in case of missed doses [64]. Wearable devices equipped with sensors can also monitor vital signs such as heart rate, blood pressure, and temperature, enabling the early detection of potential complications or side effects of BC treatments [11,65]. Following surgery, MIoT can aid in postoperative recovery and rehabilitation [66]. Prosthetic breasts embedded with MIoT sensors can monitor skin health and provide feedback on fit and comfort, facilitating a smoother transition for patients adapting to post-mastectomy changes [11,58]. Additionally, remote physical therapy and rehabilitation programs, supported by MIoT devices, ensure that BC survivors receive necessary care and guidance to regain strength and mobility from the comfort of their homes [67]. For instance, the utilization of VR for rehabilitation is well-established; however, studies examining the feasibility of VR for individualized, progressive, arm-movement practice post-breast cancer surgery are scarce [68]. Recent VR rehabilitation research in breast cancer patients mainly employs RCT designs, characterized by prospective approaches, sufficient sample sizes, and methodological rigor. Notably, VR can reduce movement-related fear and boost motivation for rehabilitation, enhancing compliance and success [69,70,71]. Additionally, VR has been shown to improve shoulder range of motion (ROM) more effectively than standard physiotherapy in postoperative rehabilitation [72]. In Wu SC et al.’s study, participants were initially skeptical about early postoperative rehabilitation [68]. However, VR played a crucial role by providing essential information, which led to positive behavioral changes. Previous research highlights the importance of understanding health improvement strategies [73], and breast cancer patients often seek detailed, real-time recovery information. VR facilitated early rehabilitation engagement by reducing fear and increasing motivation, aiming to improve shoulder and arm mobility, and was as effective as conventional physiotherapy in enhancing upper limb function for daily activities [74]. The gamified VR system alleviated fear, pain, and discomfort, enhancing rehabilitation motivation, consistent with previous studies [72,75]. The adjustable game design met patient needs, preventing the abandonment of difficult or simple movements. The system offered an engaging rehabilitation environment, promoting early and continuous rehabilitation and reducing the risk of functional impairments. However, VR accessibility is challenging, especially for home use, as not all patients can afford the equipment, limiting such interventions to in-hospital settings [68]. While the use of VR-based interventions has expanded in the rehabilitation management of breast cancer survivors, the current evidence for both immediate and long-term improvements remains limited. Future trials would benefit from using multicenter data, with larger sample sizes, longer follow-up periods, and high methodological quality [76].

Patient support and education are also areas where MIoT can play a vital role. Mobile applications and chatbots can deliver personalized information about BC, treatment options, and post-treatment care to patients, offering emotional support and connecting individuals to support groups [77]. Furthermore, telemedicine platforms integrated with MIoT devices enable remote consultations with healthcare professionals, reducing the need for frequent in-person visits during treatment. From a research perspective, MIoT-enabled wearables can collect anonymized health data from BC patients, contributing valuable insights to large-scale research studies and clinical trials [11,58]. Such data can aid researchers in identifying patterns, predicting outcomes, and developing more effective treatments. In the realm of disease progression monitoring, advanced imaging equipment with MIoT capabilities can monitor changes in breast tissue over time, facilitating the assessment of disease progression and treatment effectiveness. Finally, MIoT sensors can monitor environmental factors that may contribute to BC risk, such as pollution or exposure to harmful chemicals [78]. These data can inform public health initiatives and empower individuals to make informed decisions about their surroundings. Despite these advancements, MIoT faces challenges common to new medical technologies, such as medical supervision, insurance, and the digital divide, which require validation and large-scale clinical application [49,58,79]. Additionally, while MIoT offers promising benefits in BC care, it is essential to address privacy and security concerns to safeguard sensitive patient data [79]. Moreover, these MIoT applications should complement traditional medical assessments and be deployed under the guidance of healthcare professionals to ensure their efficacy and safety in clinical practice.

### 3.3. Digital Twins Technology

Digital twins (DTs) stand as a key technology in utilizing medical data, comprising both a physical entity and its virtual counterpart, which mutually enhance one another through iterative refinement [79]. They serve primarily as virtual representations of various physical entities, ranging from individuals to systems, crafted through the integration of advanced technologies like analytics, AI, and MIoT [80]. Professionals utilize DTs to replicate actions performed on physical entities before execution in reality, facilitating planning and optimization processes [81]. Despite their rapid adoption in industries like manufacturing, their integration into the healthcare sector has been comparatively sluggish [82,83]. However, deploying DTs in clinical care, particularly in cancer treatment, holds significant promise, owing to advancements in precision medicine and analytical capabilities [84,85,86]. With BC diagnoses entailing the consideration of diverse risk factors, DTs can potentially play a substantial role in treatment modalities [87]. By integrating heterogeneous datasets into digital replicas, advanced analytics and AI algorithms enable (near) real-time scenario simulations throughout a patient’s treatment journey [80]. Although these aspects find resonance in current literature assessments, legally approved DT-based medical solutions, especially in BC contexts, remain scarce, necessitating further real-world evidence to demonstrate their efficacy [2,88]. Furthermore, the practicality of testing multiple therapeutic interventions is challenged, underscoring the need to assess the virtual component’s effectiveness through patient-matched data [89,90]. Konopik J et al. [79] elucidated a workflow to leverage public data, particularly the BRCA dataset from TCGA, for constructing a real-world patient cohort, enriching feature matrices with variant scores and clinical features. Employing the Uniform Manifold Approximation and Projection (UMAP) clustering approach, they identified real-world examples closely matching input DT data, thereby demonstrating DT’s potential in BC research for improved patient stratification, treatment decision-making, and drug development, ultimately driving personalized medicine and optimized clinical practices [87,89,90]. Despite the proliferation of DT initiatives, detailed information on their successful implementation for complex medical data remains scarce. It is imperative that such systems are user-friendly and adaptable for healthcare professionals, who are integral to their usage and development, lacking extensive technical expertise [79]. Additionally, in their groundbreaking study, Moztarzadeh O et al. [2] demonstrated the transformative potential of digital twin technology in BC diagnosis and treatment. By using machine learning techniques and leveraging comprehensive patient data, the research team developed a digital twin capable of accurately simulating treatment options and aiding medical professionals in decision-making processes. Through the integration of digital twinning and machine learning, the study showcased promising results in accurately diagnosing BC and predicting treatment outcomes. Utilizing a dataset sourced from patients at the University Hospital Centre of Coimbra, the research team developed prediction models based on metabolic dysregulation and hyper-resistinemia—common features in cancer patients. The digital twin created in this study serves as a virtual replica of the patient, offering valuable insights into individual patient characteristics and treatment responses. While further research and development are necessary to optimize the commercial viability of digital twins in breast surgery, this study lays a solid foundation for future advancements in personalized medicine and optimized clinical practices [2]. Although promising results exist, metaverse technology has its limitations. DTs are still under development, necessitating clear instructions and ease of use for doctors, especially those lacking extensive computer science knowledge [58,85]. Challenges also arise in transferring information from the real world to the virtual system and ensuring the security of the Metaverse to prevent potential leaks of clinical data while also recognizing the importance of data protection and compliance with General Data Protection Regulation (GDPR) in the development of medical DT platforms [91,92]. By maintaining up-to-date clinical data and enabling continuous monitoring, the Metaverse could serve as an accurate and reliable platform for precise diagnoses and individualized treatments for BC patients, contributing to improvements in patient care [2]. 

### 3.4. Privacy, Security, and Ethical Considerations

Privacy and confidentiality hold significant importance for Metaverse technology. Certain medical data collected within the metaverse must adhere to existing or forthcoming privacy laws, such as the Health Insurance Portability and Accountability Act (HIPAA) in the United States [8]. In a zero-trust environment, secure computation techniques, including blockchains, play a vital role. A Metaverse system, thoughtfully designed with secure computing, can leverage raw data while safeguarding sensitive or private information. Federated learning serves as an initial step [93,94,95], offering numerous opportunities to maintain the integrity of patients’ data and to use this information to enhance clinical practice and healthcare. Patients should have control over their own data and avatars, enabled by blockchain technology, allowing them to share their digital healthcare assets as they see fit [8]. To ensure the secure and confidential handling of sensitive patient data, robust encryption and access control mechanisms can be implemented within the metaverse technology, permitting only authorized personnel to access data based on their roles and responsibilities. This approach allows for the utilization of data anonymization techniques to safeguard patient identities while facilitating medical data analysis, particularly when integrating data from various hospitals or sources. Standard interfaces and protocols can be adopted to facilitate secure and standardized data transmission and processing [94]. Compliance with GDPR [96] and ISO 27001 [97] standards pertaining to data privacy and security is crucial to ensure alignment with industry standards and best practices [95]. Initially, dataset structures and access options may be heterogeneous and hierarchical. For instance, many de-identified datasets, such as those used in various deep imaging challenges, are or will become publicly available. Conversely, certain high-value or sensitive datasets might be shared only within a consortium, healthcare system, or multi-institutional project, where implementing paywalls could be advantageous. Emerging models for data sharing include companies like Segmed, which currently sell anonymized patient data to AI developers (https://www.segmed.ai) (accessed on 2 April 2024). The vision for Metaverse supports the development of various paywall mechanisms, including subscriptions, pay-per-use, and limited trials. Non-fungible tokens also present a viable option [98]. Cybersecurity, a well-established field [99], continuously evolves to tackle new challenges. Social metaverses have already faced harassment issues, which in MeTAI could manifest as adversarial attacks on algorithms, avatar modifications, and conventional human misbehaviors. These challenges are inherent across all metaverses, and methods and rules are being developed to address them. For instance, Meta has implemented a four-foot personal zone to prevent VR groping [100]. In medical imaging, adversarial defense strategies include stabilizing image reconstruction neural networks through a combination of analytic modeling, compressed sensing, iterative refinement, and deep learning (ACID) [101,102]. Additionally, AI model explainability can be compromised by subtle input perturbations. While the primary focus is on cancer digital twins, achieving GDPR compliance and maintaining data protection in medical digital-twin platform development represents an important future objective to enhance this framework [103]. By incorporating robust data encryption, access control mechanisms, data anonymization techniques, standard interfaces and protocols, and adherence to relevant regulations and standards, the digital twin platform can emerge as an effective and secure solution for integrating and analyzing medical data from diverse sources. MeTAI (version 1.0) faces the same safety concerns as other software or hardware products, yet there is optimism that these issues can be resolved over time. The quality of evidence derived from MeTAI is expected to rapidly improve, and once validated, this digital evidence will facilitate the clinical translation of various innovations.

The incorporation of AI technologies into the Metaverse has also brought to the forefront a complex array of ethical considerations, prominently among them being the specter of bias and discrimination inherent in AI algorithms. Several studies have underscored the propensity for AI systems to perpetuate biases present in their training data, thus engendering discriminatory outcomes [104,105,106]. Complementarily, Sap M et al. [107] have shed light on how algorithmic systems, utilized for content recommendation, inadvertently reinforce stereotypes and biases, thereby curtailing the diversity of accessible information and perspectives within the Metaverse, consequently fostering echo chambers and entrenching biases [108]. Within the Metaverse, biased AI algorithms pose a significant risk of exacerbating inequality and marginalization, profoundly influencing user experiences and interactions [108]. A study on facial recognition algorithms serves as a poignant example, revealing substantial racial and gender biases manifesting in higher error rates for darker-skinned and female faces within commercial gender classification systems. Given the reliance of user avatars and virtual representations in facial recognition technology, these biases wield considerable influence, perpetuating inequality and marginalization and adversely affecting user experiences and social interactions [106]. Mitigating bias and discrimination in AI algorithms within the Metaverse demands meticulous consideration and proactive measures. One viable strategy entails ensuring the inclusivity and representativeness of training datasets. Mittelstadt BD et al. [109] stress the importance of comprehensive training data reflecting the diversity of user populations, thus precluding the underrepresentation or marginalization of specific groups. By integrating diverse perspectives during the data collection process, developers can mitigate potential biases. Furthermore, the ongoing monitoring and evaluation of AI algorithms’ performance are imperative for detecting and rectifying biases [106]. Regular audits and transparency in algorithmic decision-making processes are instrumental in identifying and rectifying biases, thereby fostering fairness and equity within the virtual realm [110,111]. The transparent reporting of AI development and deployment processes can instill trust among users, empowering them to hold AI systems accountable [112,113] Additionally, interdisciplinary collaboration is pivotal in addressing bias and discrimination in AI algorithms [114]. It necessitates concerted efforts from AI developers, platform operators, policymakers, and ethicists to collectively strive towards equitable and unbiased AI systems. Given the imperative to uphold ethical principles within the Metaverse, thereby fostering an inclusive, safe, and equitable virtual milieu [115], the establishment of guidelines and regulations is warranted to ensure responsible AI development and usage, incorporating principles of fairness, accountability, and transparency [112,116]. Addressing these ethical quandaries mandates the formulation of a robust legal and regulatory framework. Nonetheless, the current regulatory landscape for AI in virtual environments remains in its infancy [117]. Researchers are arguing for tailored regulations that address the unique ethical implications of AI technologies within the Metaverse [115,118,119]. Hence, robust legal and ethical guidelines are imperative to foster responsible AI practices within the evolving virtual sphere [106,120].

## 4. Metaverse for Training

As stated in the research by Moztarzdeh O et al., the Metaverse offers the complete anonymization of patient records [2]. This feature enables the sharing of clinical data for medical training, allowing individuals to acquire the necessary skills to perform operations on real patients. Surgeons can engage in online BC removal training exercises, preparing them adequately for real-world procedures. Research conducted by Koo H et al. illustrates how doctors can participate in simulations using HoloLens^®^ to enhance surgical outcomes and reduce errors in real-world settings [3]. This approach provides models that can be used to train surgeons, broadening their understanding of diseases. Virtual training is centered around cadaver-less anatomy education, offering immersion into a virtual environment where doctors can acquire medical knowledge while simultaneously reducing medical costs. Furthermore, in the study by Li Y et al., the advantages of Metaverse technology are highlighted [121]. This technology not only offers online training before operations but also enables telementoring during surgeries, providing real-time advice from remote specialists in rare and complicated surgical cases. According to the Champalimaud Foundation’s article, the use of HoloLens^®^ allows doctors to consult with colleagues from around the world regarding strategies for BC patients, with no latency and minimal risk of misinformation transmission [122]. The current evidence comparing surgical outcomes from metaverse-based simulations with conventional training methods remains limited. Existing studies often have small sample sizes, lack randomized controlled trials, and mainly focus on cataract surgery. Additionally, no studies have been conducted on breast cancer surgery. For instance, Antaki F et al. [123] conducted a study on RetinaVR, a virtual reality simulator for vitreoretinal surgery training using the Meta Quest 2 VR headset. Their findings showed that repeated practice on RetinaVR enhanced safety during membrane peeling and improved performance in core vitrectomy, peripheral shaving, and endolaser application. Specifically, completion times decreased by 7.67 s for core vitrectomy (*p* = 0.005), 12.02 s for peripheral shaving (*p* < 0.001), 17.92 s for membrane peeling (*p* < 0.001), and 25.68 s for endolaser application (*p* < 0.001). Safety scores improved during membrane peeling, with 1.37 fewer iatrogenic retinal touches per run (*p* = 0.003). Although similar trends were observed in all modules, not all results were statistically significant. The number of laser spots used by participants also decreased: sphere exits reduced by 5.42 times in core vitrectomy (*p* = 0.038) and by 17.00 times in peripheral shaving (*p* = 0.011). For endolaser application, 11.20 fewer laser shots were used per run to treat tears (*p* = 0.043). User experience ratings ranged from favorable to excellent in all areas. While the study did not directly demonstrate skill transfer to actual surgeries, it underscored the potential of RetinaVR as a training tool. The simulator’s performance correlated with factors such as age, sex, expertise, and experimental runs. Although further validation is needed, RetinaVR serves as a proof of concept for affordable VR surgical simulation apps, potentially revolutionizing surgical training and medical education. A Cochrane meta-analysis, which included six randomized controlled trials (RCTs) with a total of 151 postgraduate ophthalmology trainees (ranging from 12 to 60 participants per study), assessed the impact of virtual reality (VR) training on cataract surgery performance [124]. The analysis measured outcomes such as operating time, intraoperative complications, postoperative complications, supervising physician ratings, and VR simulator task ratings. The findings did not provide sufficient evidence to conclude that VR training improves cataract surgery performance compared to wet lab or conventional training methods. Although VR training shows promise as a teaching tool for cataract surgery, more rigorous, evidence-based studies are required to evaluate its effectiveness on critical outcomes, including intraoperative and postoperative complications. The current data should be interpreted cautiously due to several limitations, such as small sample sizes, imprecise descriptions of interventions and study designs, and heterogeneity in interventions and outcome assessments. Further research with robust study designs and comprehensive outcome measurements is necessary to determine the true impact of VR training on the surgical performance of postgraduate ophthalmology trainees.

## 5. Online Support Groups: Patient Coalition in the Metaverse

While the healthcare system traditionally depends on real interactions between patients and medical professionals for diagnosing and treating BC, the COVID-19 pandemic has rendered in-person appointments impossible for doctors. Consequently, technology utilization in healthcare, particularly in patient care, has surged [125]. Research conducted by Hamet P et al. indicates that the Metaverse has revolutionized the approach to standard medical practices [126].

### 5.1. Telehealth

Telehealth enhances patient diagnosis and treatment outcomes while alleviating the burden on the medical system [126]. Virtual check-ups streamline healthcare processes, enabling swift diagnosis and treatment by healthcare providers. According to the World Economic Forum in 2016, AI ranks among the top 10 most crucial emerging technologies, suggesting the potential for personalized healthcare in the foreseeable future. In their research titled “Telemedicine, Telementoring, and Telesurgery for Surgical Practice”, Jin et al. underscored telemedicine’s role as the future of healthcare delivery, offering quality care to patients [127]. Especially during events like the COVID-19 pandemic, telemedicine becomes indispensable. Notably, online visits surged from 100 to 2200 per day during restrictions, indicating the growing importance of telemedicine and its potential for further expansion. This trend benefits both patients and the healthcare sector by providing quality care in a shorter time frame.

While VR provides unprecedented immersive 3D experiences, its accessibility remains uneven. Current VR hardware and software often pose barriers due to accessibility and ergonomic challenges. A redesign could address these issues, making VR more inclusive. Early technology developments typically overlook diverse user needs, focusing on mass appeal [128]. Customizable options for vision, hearing, and motor control are needed, yet VR headsets lack built-in accessibility tools like screen readers for the visually impaired. Approximately 2.4% of the U.S. population has a visual impairment [129,130], and they are often excluded from VR usage without accommodations. Standard features in computer systems, such as adjustable font sizes and color correction tools, are absent in VR, creating additional challenges for those with visual impairments or color blindness [131,132]. Hearing impairments also present significant barriers. Globally, 10–12% of people over 15 experience moderate to severe hearing loss [132], yet VR lacks tools like live captioning or hearing aid support, found in many operating systems. Motor impairments are another concern. While desktop computers and gaming consoles offer adaptive controllers, VR systems’ compatibility with these controllers is often incomplete, complicating use. Considering hardware, ergonomic limitations in headset design affect both those with and without disabilities. Interpupillary distance (IPD) adjustments are crucial for proper visual perception and preventing simulator sickness, yet many headsets offer limited IPD ranges, insufficient for all users [133,134]. Additionally, most commercial VR headsets, weighing 450–650 g, are front-heavy, causing discomfort during extended use [135,136]. To overcome these limitations, particularly for older patients who face compounded challenges related to vision, hearing, and mobility, further research is essential to optimize weight distribution for enhanced ergonomic comfort in VR systems.

### 5.2. Online Genetic Consulting

**Figure 1:**
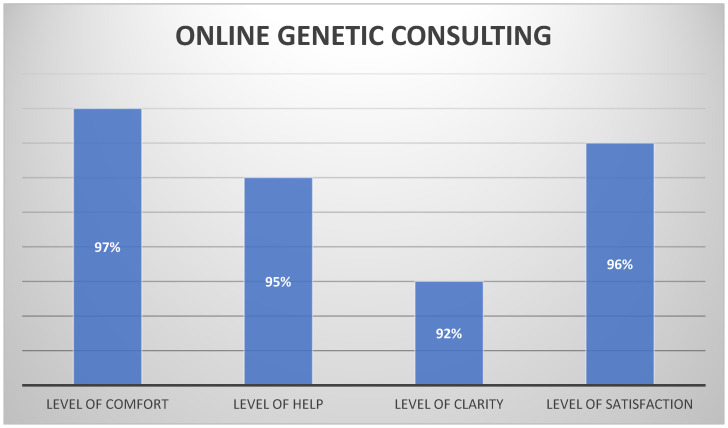
Outcomes of virtual reality-based genetic counseling sessions.

Yoo B et al. provided a review on whether a Metaverse platform could serve as an alternative service-delivery model for group genetic counseling [10]. The study involved 131 participants categorized into three groups: patients, families of patients, and individuals interested in expanding their knowledge about BC. According to the results, 95% of all attendees reported an increased understanding of hereditary BC following the group genetic counseling session, with a significant improvement in knowledge noted post-counseling. Among all participants, 87.8% indicated that the Metaverse session was more effective than face-to-face sessions, with 93.1% expressing a willingness to recommend group genetic counseling using the Metaverse platform to family members or acquaintances. Additionally, 92.4% of participants expressed a desire to participate in other educational sessions using a Metaverse platform in the future. The primary advantages cited by participants included the online location, flexibility of scheduling, and the opportunity to interact with individuals sharing similar interests within a virtual space. Conversely, the main disadvantages identified were an unstable internet connection, difficulty in maintaining concentration throughout the entire session, and challenges in receiving answers to private questions. Hynes J et al. [137], in their research, proposed that group information sessions followed by individual mini-sessions facilitated the delivery of high-quality genetic counseling while reducing wait times. The quality of these sessions was evaluated through an anonymized questionnaire, with 97% of participants strongly agreeing that they felt comfortable during group sessions, 95% finding the appointment helpful, and 92% indicating that the explanation of cancer genetics was clear, as shown in Figure 1. This suggests that online sessions not only benefit participants but also save time. Furthermore, Benusiglio P et al. [138] included a total of 210 patients in their article on group counseling and demonstrated that this counseling style allowed for the examination of more patients within a four-hour timeframe compared to private sessions. Satisfaction and knowledge levels were evaluated, with 96% of participants reporting a good level of genetic understanding. Satisfaction levels were high, with 41 out of 43 participants giving the maximum score on the questionnaire. However, despite the promising results, it is essential to acknowledge the limitations of the existing literature. Many studies lack a robust methodology and rely on self-report measures, which may introduce bias and limit the generalizability of findings. Furthermore, the majority of studies are observational or retrospective in nature, highlighting the need for future randomized controlled trials (RCTs) and comparative studies to better understand the comparative effectiveness of online genetic counseling compared to traditional face-to-face counseling. Moreover, future research endeavors should prioritize the development of standardized outcome measures to assess the efficacy of online genetic counseling accurately. By employing validated instruments and longitudinal follow-ups, researchers can ensure the validity and reliability of their findings, thus advancing our understanding of the potential benefits and limitations of virtual counseling platforms.

In conclusion, online genetic consulting represents an innovative approach to educating individuals about BC. The Metaverse platform could be utilized for telegenetic counseling, thereby enhancing the quality of patient care and our understanding of BC genetics.

### 5.3. Online Patient Therapies

**Figure 2:**
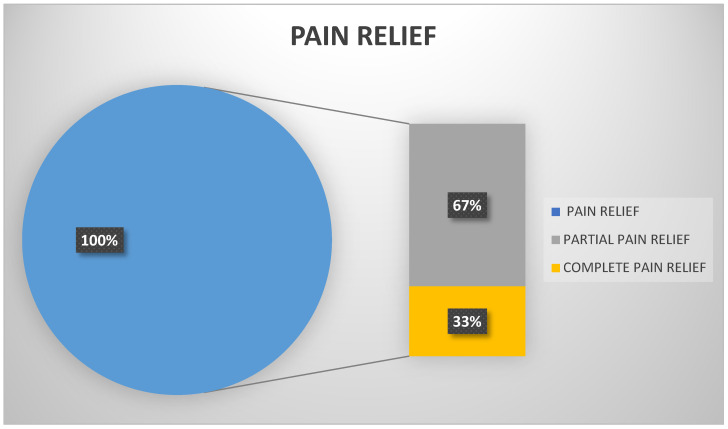
Effectiveness of VR in pain relief.

Telemedicine also enables patients to receive support during breast therapy, thereby reducing their anxiety [139]. The use of the HoloLens^®^ allows patients to immerse themselves in a virtual world and engage in online sessions amidst natural surroundings, helping them to calm down and lower their stress levels. VR shows promise as a tool for therapy to alleviate pain and anxiety. The official paper from The White House titled “Epidemic: Responding to America’s Prescription Drug Abuse Crisis” highlights a dramatic increase in the use of opioids to treat pain conditions [140]. VR has emerged as an effective alternative to opioid analgesics, even in cases of high levels of pain such as burn pain [141,142]. After analyzing the survey results from Jones T et al., it was found that 100% of participants reported a decrease in pain to some degree between pre-session pain and during-session pain. Furthermore, 33% of participants reported complete pain relief during VR sessions [143], as shown in Figure 2.

For comparison, morphine typically reduces pain by approximately 30% [144]. Additionally, participants rated their engagement in the virtual world on a scale from 0 to 10, with an average rating of 8.4. Similarly, they were asked to rate the realism of the virtual world on the same scale, yielding an average rating of 6.5. Regarding side effects, most studies examining the use of VR in the treatment of breast cancer patients did not report significant cybersickness symptoms, suggesting that technological advancements have mitigated these adverse effects [76,145,146]. Cybersickness encompasses symptoms and adverse side effects experienced during or after VR immersion, including nausea, headache, dizziness, vomiting, eyestrain, fatigue, disorientation, ataxia, pallor, dry mouth, and sweating [147,148]. In their study, Chirico A et al. utilized the Virtual Reality Symptom Questionnaire (VRSQ) to assess cybersickness symptoms, finding that apart from minor concentration difficulties, symptoms such as nausea, dizziness, headache, drowsiness, and eyestrain were reported by less than 20% of patients [149,150]. A systematic review and meta-analysis by Caserman P et al. [147] revealed that advancements in VR technology and contemporary VR head-mounted displays (HMDs) significantly reduced the incidence of cybersickness (*p* < 0.001), though some symptoms persist. They identified sensory mismatch and perceived motion as the primary contributors to cybersickness in VR environments. Additionally, the use of VR as a distraction intervention has been shown to alleviate symptoms such as pain, stress, anxiety, depression, fatigue, and nausea, among others [151]. Numerous studies have demonstrated that VR can significantly contribute to patient empowerment and education, rehabilitation, the management of cancer-related symptoms, psychiatric disorders, and the mitigation of treatment side effects [76,145,152,153,154]. Nonetheless, this technology is not without its limitations, which include cybersickness, discomfort, user resistance, and the high cost of equipment [130,136,155]. Furthermore, VR has been found to significantly reduce pain and anxiety. Telemedicine, through virtual sessions that allow patients to engage with nature, serves as an effective tool to enhance well-being prior to breast-conserving therapy and to aid in post-operative recovery [139,143,156].

### 5.4. Financial Awareness Support

Not only does the Metaverse support therapy, but it also serves as a platform to raise awareness about BC. The foundation known as Susan G. Komen was established in 1982 and has since grown into an exemplary safe space for sharing doubts and questions among BC patients, inspiring and expanding over the years [157]. It spreads awareness globally, fostering a sense of community and support among women. Susan G. Komen has created a united space for women to share experiences, seek support, and find answers to their questions. Over the past five years, Susan G. Komen has allocated more than 80 cents of every dollar directly towards research, community-based health programs, education, and supportive initiatives. In 2023 alone, over 3 million women received BC education, with USD 19 million invested in research and interventions to address BC disparities. Additionally, the Susan G. Komen platform provided 42,000 services through the Patient Care Center, marking a remarkable 64% growth compared to the previous year. Analyzing these achievements underscores the significant benefits such a growing platform offers to BC patients. Thus, the Susan G. Komen initiative has partnered with the Metaverse to provide financial support through contributions to women in need—an initiative that not only raises awareness but also aids those requiring assistance [158]. 

## 6. Dr. Meta

Recently, a new platform was described in the article written by Kim S et al. titled “Multidomain Metaverse Cancer Care Platform: Development and Usability Study” [159]. Dr. Meta is a platform designed in South Korea to enhance the healthcare system, showcasing the Metaverse in a new version with unlimited possibilities. It functions as a medical conference venue, educational platform, and online patient support page. Doctors can access the virtual world to exchange medical information and support one another, while students and young doctors can receive education through online lectures and immersive clinical practice. Additionally, the platform serves as a valuable research environment, with online storage of medical information utilized to prepare medical articles for future diagnostic and treatment guidelines. Dr. Meta is not only beneficial for doctors but also for patients, offering virtual services and the ability to contact doctors or other patients for support; this is particularly advantageous for patients in remote areas. The immersive world encompasses various sub-platforms, fostering collaboration with health professionals. Following the immersive experience, patients and doctors completed questionnaires. Seventy-two percent of participants found Dr. Meta to be an interesting and immersive platform, with 59% expressing a desire to continue using it in the future. Sixty percent indicated they would recommend it to others. Participants emphasized its potential to benefit the community medical field, its exciting and immersive educational system, and its liveliness compared to face-to-face or previous online training. However, one of the disadvantages of the HoloLens^®^ is its weight, which can strain participants’ backs, and sometimes the experiences feel unnatural. Therefore, further development of the platform is necessary to address these issues. The new graphic design aims to enhance immersion in the virtual world. Additionally, with technological advancements, future iterations of the Metaverse could operate on different devices, minimizing the inconvenience of wearing the HoloLens^®^. While the technology is in its early-stage pilot and requires proper security measures as it grows, the sophisticated Dr. Meta healthcare platform offers a fresh approach, consolidating the benefits of the Metaverse in one place. Through continuous upgrades, Dr. Meta shows potential to contribute to advancements in the healthcare system, potentially expanding into an international medical platform beyond initial projections.

## 7. Future Research

More research is needed to investigate the impact of the metaverse in clinical settings and gather more accurate data. As the technology continues to evolve, future updates to the metaverse can enhance the experiences of virtual and augmented reality (AR). Currently, there are several limitations, including the significant costs associated with hardware, software, and maintenance, which many hospitals may find prohibitive. Integrating the metaverse with existing healthcare systems and workflows also presents substantial challenges. Understanding the economic implications of integrating new technologies into healthcare is crucial for assessing their feasibility and sustainability. To date, no studies have specifically addressed the cost analysis of Metaverse technology in breast cancer care, highlighting a significant gap in the literature. We emphasize this deficiency and call for future research to conduct comprehensive cost–benefit analyses of Metaverse technology in routine breast cancer care. By addressing this need, studies elucidating the financial aspects should be encouraged, aiding healthcare providers in making informed decisions regarding the adoption of such technologies. Furthermore, VR technology may encounter latency issues, requiring technical support and potentially hindering doctors’ effectiveness. In surgical settings, latency could pose risks to patient safety. Developing high-quality, medically accurate VR systems is challenging and costly, particularly when creating content for specific procedures or training simulations. Additionally, rigorous regulations and the validation of Metaverse applications are necessary to ensure their safety, efficacy, and accuracy. User experience is another limitation, with variations depending on hardware quality, software design, and personal preferences. Ensuring an immersive experience free of physical discomfort is crucial. Currently, limited evidence supports the effectiveness of the metaverse and VR technology in healthcare. Further research is needed to evaluate clinical outcomes, and patient satisfaction. Another notable limitation is the lack of existing research on the long-term reliability and maintenance requirements of Metaverse technology in clinical settings. Future research should also focus on evaluating system updates, technical support, and long-term operational costs to ensure sustainable integration of Metaverse technology in healthcare environments. Despite these limitations, ongoing advancements in VR technology and the metaverse hold the potential to address many of these challenges and enable the full benefits of the metaverse in medicine.

## 8. Conclusions

The Metaverse demonstrates potential advantages over the traditional healthcare system in diagnosing and treating BC. This evolving technology enables simulations of BC operations, video conferences, and scientific research [3]. We suggest this platform could have a positive impact on the healthcare system, potentially enhancing productivity and efficiency. The Metaverse presents an opportunity for healthcare professionals to engage with patients more immersively, potentially leading to improved patient satisfaction. It facilitates transmission of clinical data, allowing for analysis and diagnosis, leading to potential treatment optimization and improved care. Our analysis of various studies suggests potential benefits for patients, which may result in reduced healthcare costs and improved treatment outcomes [8]. Virtual environments like Digital Twins enable the simulation of diagnoses and prediction of complications before they occur. Additionally, virtual worlds aid patients in the recovery process after operations through teleconsulting, providing mental support, and promoting awareness [7,160]. In our view, this technology has the potential to enhance the medical system, making it more efficient, cost-effective, reliable, and safer. However, further research and practical implementation are necessary to fully understand its impact on BC therapy.

## Data Availability

Not applicable.
